# Aspirin Modulates Innate Inflammatory Response and Inhibits the Entry of *Trypanosoma cruzi* in Mouse Peritoneal Macrophages

**DOI:** 10.1155/2014/580919

**Published:** 2014-06-19

**Authors:** Aparecida Donizette Malvezi, Rosiane Valeriano da Silva, Carolina Panis, Lucy Megumi Yamauchi, Maria Isabel Lovo-Martins, Nagela Ghabdan Zanluqui, Vera Lúcia Hideko Tatakihara, Luiz Vicente Rizzo, Waldiceu A. Verri, Marli Cardoso Martins-Pinge, Sueli Fumie Yamada-Ogatta, Phileno Pinge-Filho

**Affiliations:** ^1^Laboratório de Imunopatologia Experimental, Departamento de Ciências Patológicas, Centro de Ciências Biológicas, Universidade Estadual de Londrina, 86057-970 Londrina, PR, Brazil; ^2^Laboratório de Biologia Molecular de Microrganismos, Departamento de Microbiologia, Centro de Ciências Biológicas, Universidade Estadual de Londrina, 86057-970 Londrina, PR, Brazil; ^3^Instituto Israelita de Ensino e Pesquisa Albert Einstein, 056510-901 São Paulo, SP, Brazil; ^4^Departamento de Ciências Patológicas, Centro de Ciências Biológicas, Universidade Estadual de Londrina, 86057-970 Londrina, PR, Brazil; ^5^Departamento de Ciências Fisiológicas, Centro de Ciências Biológicas, Universidade Estadual de Londrina, 86057-970 Londrina, PR, Brazil

## Abstract

The intracellular protozoan parasite *Trypanosoma cruzi* causes Chagas disease, a serious disorder that affects millions of people in Latin America. Cell invasion by *T. cruzi* and its intracellular replication are essential to the parasite's life cycle and for the development of Chagas disease. Here, we present evidence suggesting the involvement of the host's cyclooxygenase (COX) enzyme during *T. cruzi* invasion. Pharmacological antagonist for COX-1, aspirin (ASA), caused marked inhibition of *T. cruzi* infection when peritoneal macrophages were pretreated with ASA for 30 min at 37°C before inoculation. This inhibition was associated with increased production of IL-1*β* and nitric oxide (NO^∙^) by macrophages. The treatment of macrophages with either NOS inhibitors or prostaglandin E_2_ (PGE_2_) restored the invasive action of *T. cruzi* in macrophages previously treated with ASA. Lipoxin ALX-receptor antagonist Boc2 reversed the inhibitory effect of ASA on trypomastigote invasion. Our results indicate that PGE_2_, NO^∙^, and lipoxins are involved in the regulation of anti-*T. cruzi* activity by macrophages, providing a better understanding of the role of prostaglandins in innate inflammatory response to *T. cruzi* infection as well as adding a new perspective to specific immune interventions.

## 1. Introduction

*Trypanosoma cruzi *is an intracellular protozoan parasite causing Chagas disease, which affects millions of people in Latin America. During the acute inflammatory phase of the* T. cruzi *infection, high-level expression of inducible nitric oxide synthase (iNOS) [1], proinflammatory cytokines [2], and arachidonic acid- (AA-) derived lipids such as leukotrienes, lipoxins (LXs), H (P) ETEs, prostaglandins, and thromboxane is prevalent [3, 4]. In the early* T. cruzi* infection, nitric oxide (NO^∙^) and arachidonic acid metabolites could be attributed to resistance, but later on to tissue damage [4].

Prostaglandins (PGs) are oxygenated lipid mediators formed from the *ω*6 essential fatty acid, arachidonic acid (AA). The committed step in PG biosynthesis is the conversion of AA to PG H_2_ (PGH_2_), catalyzed by either PG endoperoxide H synthase-1 or -2, commonly known as cyclooxygenase-1 (COX-1) and cyclooxygenase-2 (COX-2), respectively [5, 6]. Both COX-1 and COX-2 are nonselectively inhibited by nonsteroidal anti-inflammatory drugs (NSAIDs) such as aspirin and ibuprofen, whereas COX-2 activity is selectively blocked by COX-2 inhibitors called coxibs (e.g., celecoxib) [7, 8]. The relevance of these enzymes and the bioactive lipids that they produce are not well understood in parasitic disease, although the role of eicosanoids in the pathogenesis of Chagas disease is becoming more defined [3]. Pharmacological antagonists of COX-1 (aspirin, ASA), COX-2 (celecoxib), or both (indomethacin) have been found to increase mortality and parasitemia (parasite load in peripheral blood and cardiac tissue) regardless of which mouse or* T. cruzi* strains were used [9–13]. Moreover, evidence suggests that administration of NSAIDs may enhance mortality in chagasic patients [12]. Conversely, others have found that inhibition of PG synthesis/release reduces parasitemia and extends survival of mice infected with* T. cruzi* [14–17]. This was often associated with a decrease in the levels of circulating inflammatory cytokines (such as TNF-*α*, IFN-*γ*, and IL-10) [16]. More recently, treatment with ASA during chronic infection was found to be beneficial with no increase in mortality and substantial improvement in cardiac function [13]. Additionally, the protective effect of ASA could be mediated by the synthesis of 15-epi-lipoxin A_4_ (15-epi-LXA_4_) [18].

Given the increasing interest in the role of eicosanoids in* T. cruzi* infection, we decided to investigate the effect of prostaglandin synthesis inhibition with ASA on inflammatory response and macrophage invasion by* T. cruzi*.

## 2. Material and Methods

### 2.1. Animals

Six- to eight-week-old BALB/c female and male mice were supplied by the Multi-Institutional Center for Biological Investigation, State University of Campinas, Brazil. Mice were maintained under standard conditions in the animal house of the Department of Pathological Sciences, Center for Biological Sciences, State University of Londrina. Commercial rodent diet (Nuvilab-CR1, Quimtia-Nuvital, Colombo, Brazil) and sterilized water were available* ad libitum*.

All animal procedures were performed in accordance with the guidelines of the Brazilian Code for the Use of Laboratory Animals. The protocols were approved by the Internal Scientific Commission and the Ethics in Animal Experimentation Committee of Londrina State University (Approval Number: CEEA 5492.2012.22).

### 2.2. Parasites

*T. cruzi* Y [19] was maintained by weekly intraperitoneal inoculation of Swiss mice with 2 × 10^5^ trypomastigotes. To conduct our experiments, blood from previously infected mice was obtained by cardiac puncture without anticoagulant. The blood was centrifuged at 1,500 ×g for 1 min and allowed to stand at 37°C for 60 min. The supernatant serum containing most of the* T. cruzi* was centrifuged at 1,200 ×g for 15 min. The sediment was resuspended in 1 mL of RPMI 1640 medium (GIBCO, Gran Island, NY) containing 10% inactivated fetal bovine serum (FBS), 100 units of penicillin, and 100 *μ*g streptomycin (GIBCO, Gran Island, NY).

Trypomastigotes were derived from the supernatant of* T. cruzi*-infected LLC-Mk2 culture cells (ATCC CCL-7; American Type Culture Collection, Rockville, MD) grown in RPMI 1640 medium containing 10% inactivated fetal bovine serum (FBS), 40 *μ*g mL^−1^ gentamicin, 100 units of penicillin, and 100 *μ*g streptomycin (GIBCO, Gran Island, Y). Subconfluent cultures of LLC-Mk2 were infected with 5 × 10^6^ trypomastigotes. Free parasites were removed after 24 h and cultures were maintained in 10% FBS-RPMI 1640. Five days postinfection, free trypomastigote forms could be found in the cell supernatants.

### 2.3. Macrophage Culture

Mice were inoculated intraperitoneally with 2 mL of 5% thioglycollate and, 4 days later, the elicited cells from the peritoneal exudates were harvested in cold PBS. Mouse peritoneum was washed with 5 mL ice-cold, serum-free RPMI. Peritoneal cells from 3–6 mice were pooled and left to adhere in complete medium (RPMI, 2 mM glutamine, 1 mM sodium pyruvate, 40 *μ*g mL^−1^ gentamicin, and 10 mM HEPES) for 24 h in 24-well plates at 2 × 10^5^ cells/well. Each suspension of pooled peritoneal cells was plated in triplicate wells. Then, nonadherent cells were washed away and adherent cells received complete medium. The macrophages were plated onto 13 mm round glass coverslips and washed in warm phosphate-buffered saline (PBS) before the interaction assays. In addition, 2.0 × 10^5^ macrophages were plated onto 96-well dishes. One set of plates was used to quantify IL-1*β* and the other set for NO^∙^ detection.

### 2.4. Treatment of Macrophages with Drugs and Macrophage Invasion Assay

Before the experiments, peritoneal macrophages previously washed were incubated for 30 min at 37°C in a 5% CO_2_ atmosphere in the presence of different concentrations of ASA (2.5 mM, 1.25 mM, and 0.625 mM) to test its effect on internalization of the parasite into the host cell. After incubation, the medium containing ASA was removed, and macrophages were allowed to interact with trypomastigote forms added in a ratio of 5 parasites per cell. The interaction was allowed to proceed for 2 h, at 37°C in a 5% CO_2_ atmosphere. The cells were then washed three times, fixed with Bouin's fixative, stained with Giemsa (Merck) stain, and observed with a light microscope at 1000x magnification. Other treatments included incubation with aminoguanidine (1 mM) or L-NAME (1.0 mM) for 60 min at 37°C with or without ASA.

The internalization index was calculated by multiplying the percentage of infected cells by the mean number of parasites per infected cell [20]. All internalization indices were normalized. Experiments were performed in triplicate, and six independent experiments were completed. All experiments included untreated, infected peritoneal macrophages as controls. The quantification was carried out using light microscopy where a total of 500 cells were randomly counted. The viability of the cells obtained from the cultures before and after incubation experiments was determined using MTT (Sigma-Aldrich) assay, showing the mitochondrial activity of living cells. The culture medium was aspirated, and MTT (0.5 mg mL^−1^) was added to the cells prior to incubation at 37°C for 4 h. The supernatant was aspirated and dimethyl sulfoxide (Sigma-Aldrich) was added to the wells. Insoluble crystals were dissolved by mixing and the plates were read using a BioRad multiplate reader (Hercules, CA), at a test wavelength of 570 nm and a reference wavelength of 630 nm.

### 2.5. Nitrite Measurements

Production of nitric oxide (NO^∙^) was determined by measuring the level of accumulated nitrite, a metabolite of NO^∙^ in the culture supernatant using Griess reagent (Sigma-Aldrich). After 24 h of treatment with ASA (0.625 mM), the culture supernatants were collected and mixed with an equal volume of Griess reagent in 96-well culture plates and incubated at room temperature for 10 min. The absorbance was measured at 540 nm and nitrite concentrations were calculated by reference to a standard curve generated by known concentrations of sodium nitrite.

### 2.6. Immunocytochemistry Labeling for iNOS

Immunocytochemistry for iNOS was performed on coverslip-adherent cells using the labeled streptavidin biotin method with a LSAB KIT (DAKO Japan, Kyoto, Japan) without microwave accentuation. The coverslips were incubated with 10% Triton X-100 solution for 1 h, washed 3 times in PBS, and treated for 40 min at room temperature with 10% BSA. The coverslips were then incubated overnight at 4°C with the primary antibody (anti-iNOS rabbit monoclonal antibody diluted 1 : 200, BD Biosciences, catalog number 610599), followed by secondary antibody treatment for 2 h at room temperature. Horseradish peroxidase activity was visualized by treatment with H_2_O_2_ and 3,3′-diaminobenzidine (DAB) for 5 min. At the last step, the sections were weakly counterstained with Harry's hematoxylin (Merck). For each case, negative controls were prepared by omitting the primary antibody. Intensity and localization of immune reaction against primary antibody used were examined on all coverslips using a photomicroscope (Olympus BX41, Olympus Optical Co., Ltd., Tokyo, Japan). For the image analysis study, photomicroscopic color slides of representative areas (magnification, 40x) were digitally acquired. After conversion of the images into grey scale (Adobe Photoshop) iNOS-positive pixels and total pixels became thresholds and were processed by Image J program. Positive immunostained area was calculated as the proportion of positive pixels to total pixels (%).

### 2.7. ELISA for IL-1*β*

Culture supernatants from peritoneal macrophages in 96-well plates treated with ASA (0.625 mM) or untreated, either infected or not infected with* T. cruzi*, were incubated for 24 h. Levels of IL-1*β* in 100 *μ*L medium were measured by commercial ELISA kits (Ready-SET-Go! eBioscience, San Diego, CA), according to the manufacturer's instructions.

### 2.8. Statistical Analysis

The statistical analysis was conducted using one way ANOVA with Bonferroni's multiple comparison test. Values are presented as ± standard error of mean. The results were considered significant when *P* < 0.05. Statistical analysis was performed using the GraphPad Prism 5.0 computer software application (GraphPad Software, San Diego, CA, USA).

## 3. Results

### 3.1. Aspirin Inhibits* T. cruzi* Entry into Macrophages

**Figure 1:**
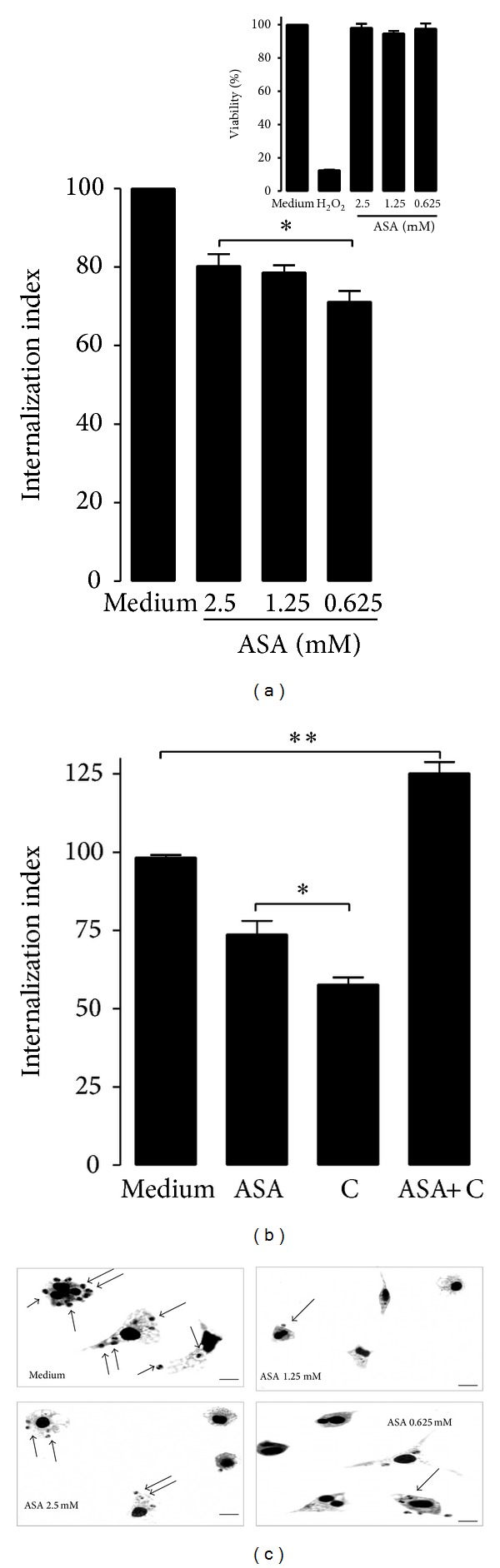
Aspirin (ASA) impairs* Trypanosoma cruzi* internalization by peritoneal macrophages. (a) Internalization index of the interaction process between macrophages treated for 30 minutes with increasing concentrations of ASA (0.625, 1.25, and 2.5 mM) and exposed to* T. cruzi* (Y strain). After treatment with ASA, peritoneal macrophages interacted with 5 : 1 trypomastigotes for 2 hours, after which they are washed, fixed with Bouin's fixative, and stained with Giemsa. Quantification was carried out under a light microscope where the number of intracellular parasites was counted in a total of at least 500 cells. MTT assay to measure cell viability in macrophages after treatment with ASA at 0.625 to 2.5 mM concentrations. H_2_O_2_ (1000 *μ*M) was used as negative control (insert). Values are the means ± standard error of mean of 10 experiments or two experiments (MTT assay). *P < 0.0001 for a comparison with infected cells cultured in medium alone. (b) The combined effect of celecoxib and aspirin on the entry of* T. cruzi* into phagocytic cells. Macrophages were treated for 30 minutes with celecoxib (0.625 mM) and ASA (0.625 mM) exposed to* T. cruzi* as describe above. Results are the mean ± standard error for triplicate determinations and are representative of three independent experiments. *P < 0.0001, **P < 0.001 for a comparison with infected cells cultured in medium alone. (c) Light microscopy observations confirm* T. cruzi* inhibition invasion of ASA treated cells. Observation after Giemsa staining by light microscopy of the interaction process between peritoneal macrophages treated (or not) with different concentration of ASA and exposed to trypomastigotes forms of* T. cruzi*. The black arrows indicate internalized parasites. Bars = 10 *μ*m.

To determine whether COX-derived mediators are involved in* T. cruzi* entry into host cells, cells were treated with increasing amounts of ASA for 30 min and after treatment, the medium containing ASA was removed before macrophage invasion assay in order to guarantee that ASA affected only the host cell and not the parasites. After 2 h of incubation with parasites, which provides sufficient time for them to enter into macrophages, the free parasites were removed. Aspirin irreversibly inhibits COX-1 by acetylation of a single serine residue on the enzyme [21] and this inactivation persists, that is, ≥24 hours. In some cases, the medium with increasing ASA concentrations was added every 24 hours until the end of the* T. cruzi* infection period (7 days, Figure 4(d)).  Figure 1(a) shows that ASA markedly inhibited the internalization of trypomastigote by macrophages at all concentrations tested (*P* < 0.0001). Thus, PGE_2_ synthesis inhibition using ASA improves macrophage response against* T. cruzi* infection. The cytotoxicity of ASA in macrophages was evaluated by MTT assay (Figure 1(a), insert). ASA did not induce cell death, as the concentrations of ASA used in all experiments reported were too low to cause cytotoxicity [22]. Light microscopy observations confirm* T. cruzi* inhibition invasion of ASA treated cells (Figure 1(c)).

### 3.2. COX-2 Inhibition Together with Aspirin Restores the Infectivity of Trypomastigotes in Peritoneal Macrophages

We examined the combined effect of celecoxib (COX-2 selective inhibitor) and ASA on the entry of* T. cruzi* into phagocytic cells. Peritoneal macrophages were incubated separately, with or without drugs (ASA and celecoxib) either alone or in combination.  Figure 1(b) shows that the drugs in combination significantly (*P* < 0.01) restored the invasive capacity of trypomastigotes. None of the drugs (ASA, celecoxib, or their combination) showed cytotoxicity against uninfected peritoneal macrophages (data not shown). These results indicate that the enzyme activities of COX-1 and COX-2 favor invasion, but simultaneous inhibition of both activities also favors parasite invasion, probably by allowing a new panorama of eicosanoids production.

### 3.3. Aspirin Decreases Trypomastigotes Release to Culture Supernatants from* T. cruzi*-Infected Macrophages

Four days postinfection, macrophages began releasing trypomastigotes into the supernatant (Figure 4(d)). Trypomastigotes release to culture supernatants from* T. cruzi*-infected macrophages was diminished by ASA (0.625 mM). This result is in agreement with previous reports, showing that aspirin and other COX inhibitors decrease* T. cruzi* infection* in vitro* [14–16, 23].

### 3.4. Aspirin Inhibits* T. cruzi* Entry into Macrophages via ALX

**Figure 2:**
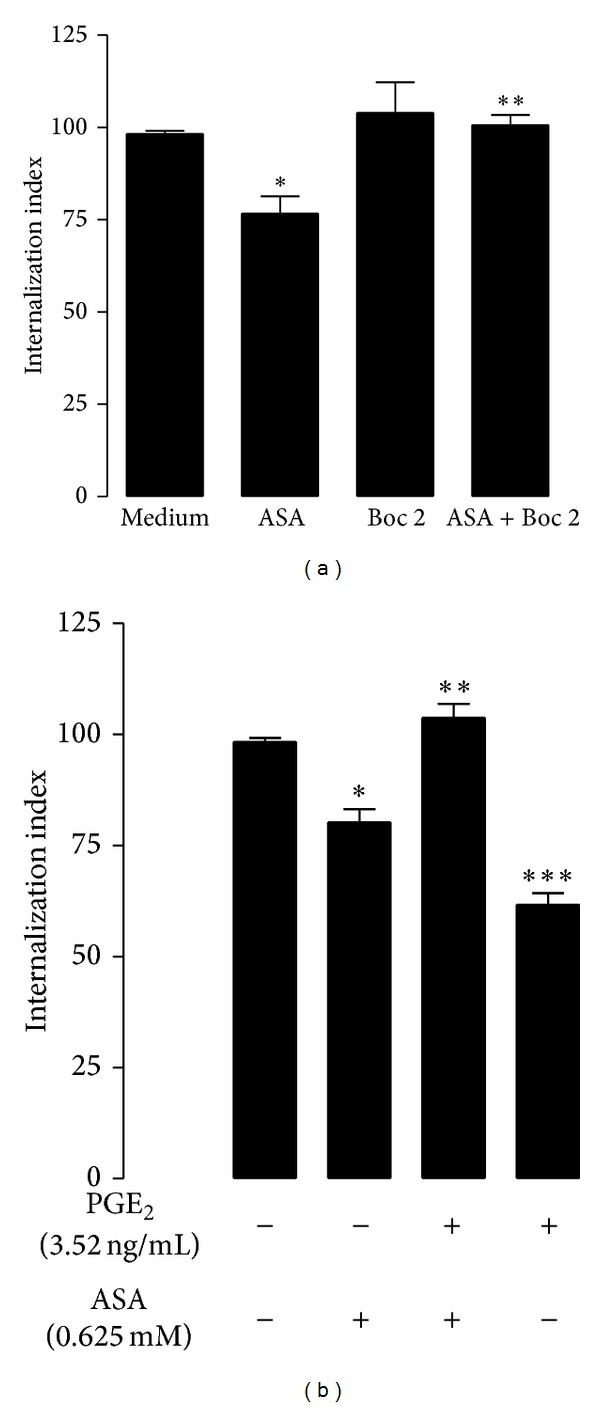
The effect of ASA on the entry of* T. cruzi* into macrophages was prevented by Boc-2. (a) Internalization index of the interaction process between macrophages treated for 30 minutes separately either with or without drugs (0.625 *μ*M ASA or 100 *μ*M Boc-2). Results are the mean ± standard error for triplicate determinations and are representative of two independent experiments. *P < 0.01, for a comparison with infected cells cultured in medium alone. **P < 0.001, for a comparison with infected cell treated with ASA. (b) PGE_2_ restores aspirin effect on* T. cruzi* entry into macrophages. Macrophages were treated for 30 minutes separately either with or without PGE_2_ (35.2, 3.52, or 0.35 ng mL^−1^) alone or in combination (ASA 0.625 mM + PGE_2_ 3.52 ng mL^−1^). Results are the mean ± standard error and are representative of two independent experiments. *P < 0.001 for a comparison with infected cells cultured in medium alone. **P < 0.05 for comparison with infected cell treated with ASA or PGE_2_ alone. ***P < 0.05 for a comparison with infected cells cultured in medium alone.

Lipoxins (LXs) are endogenous lipid mediators with potent anti-inflammatory and proresolving actions [24, 25]. Native LXs and their stable analogues exert their biological effects by binding to a G-protein-coupled receptor, denoted as ALX [26, 27]. The effect of ASA has been associated, in part, to a switch to the AA pathway linked to the acetylation of the COX-2 isoenzyme. This reaction enables COX-2 to synthesize LXs as 15-epi-LXs (15-epi-LXA4) [22]. To evaluate the effect of ASA-triggered LXs on the entry of* T. cruzi* into phagocytic cells, peritoneal macrophages were incubated separately either with or without drugs (ASA or Boc-2, a specific antagonist of the 15-epi-LXs) either alone or in combination. The inhibitory effect of ASA on the entry of* T. cruzi* into macrophages was prevented by Boc-2 demonstrating that the effect of ASA on the entry of* T. cruzi* into macrophages could be mediated by the synthesis of 15-epi-LXA4. Furthermore, Boc-2 did not have any effect on the entry of* T. cruzi* into macrophages when used alone (Figure 2(a)).

### 3.5. PGE_2_ Restores Aspirin Effect on* T. cruzi* Entry into Macrophages

Figure 2(b) (insert) shows that treatment of macrophages with high concentrations of PGE_2_ causes inhibition of* T. cruzi* entry into macrophages. When PGE_2_ (3.52 ng mL^−1^) was added alone or in combination with ASA, the effect of ASA was inhibited, indicating that PGE_2_ is involved in the internalization of* T. cruzi* trypomastigotes into peritoneal macrophages.

### 3.6. Aspirin Modulates Innate Inflammatory Response of Macrophages Infected with* T. cruzi*

**Figure 3:**
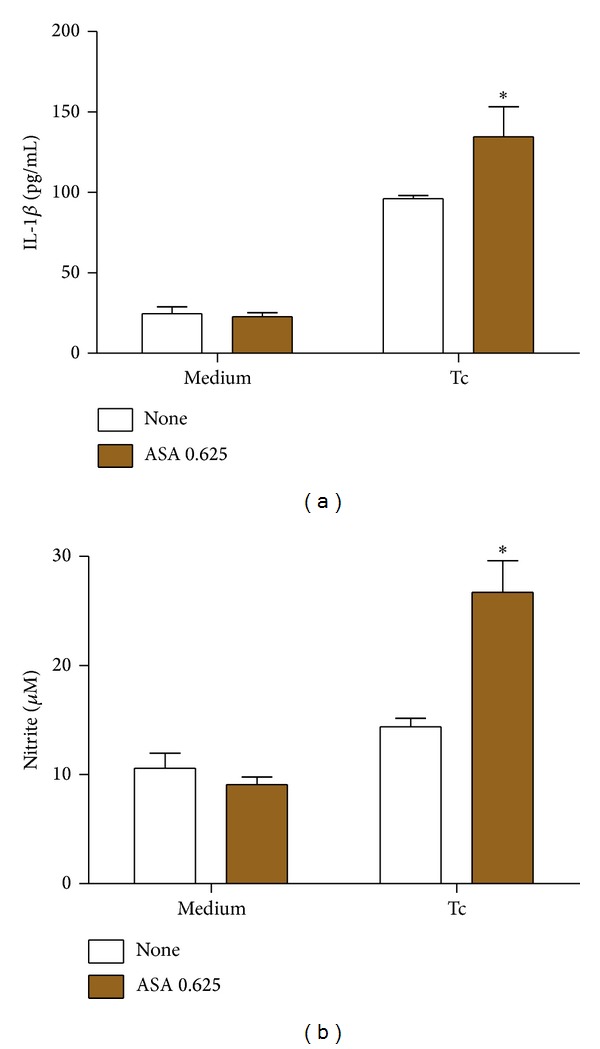
Effects of ASA upon IL-1*β* and nitrite production in* T. cruzi*-infected macrophages. Macrophages were treated for 30 minutes with ASA (0.625 mM) and exposed to* T. cruzi* (Y strain). After treatment with ASA, cells interacted with 5 : 1 trypomastigotes for 2 hours, after which they are cultured at 37°C in 5% CO_2_ during 24 h. Afterwards, IL-1*β* (a) and nitrite (b) levels in supernatant were measured with a specific enzyme-linked immunosorbent assay and by Griess reaction, respectively. Results are the mean ± standard error for duplicate determinations and are representative of four independent experiments. *P < 0.05 for a comparison with cell culture in medium alone.

The internalization of* T. cruzi* into macrophages stimulated the release of IL-1*β* while ASA increased IL-1*β* production by infected macrophages (Figure 3(a)). The effect of ASA on NO^∙^ production was evaluated by detection of nitrite in* T. cruzi*-infected macrophage supernatants using Griess reaction (Figure 3(b)). NO^∙^ production in macrophages was stimulated by* T. cruzi* and was also increased by prior treatment of macrophages with ASA.

**Figure 4:**
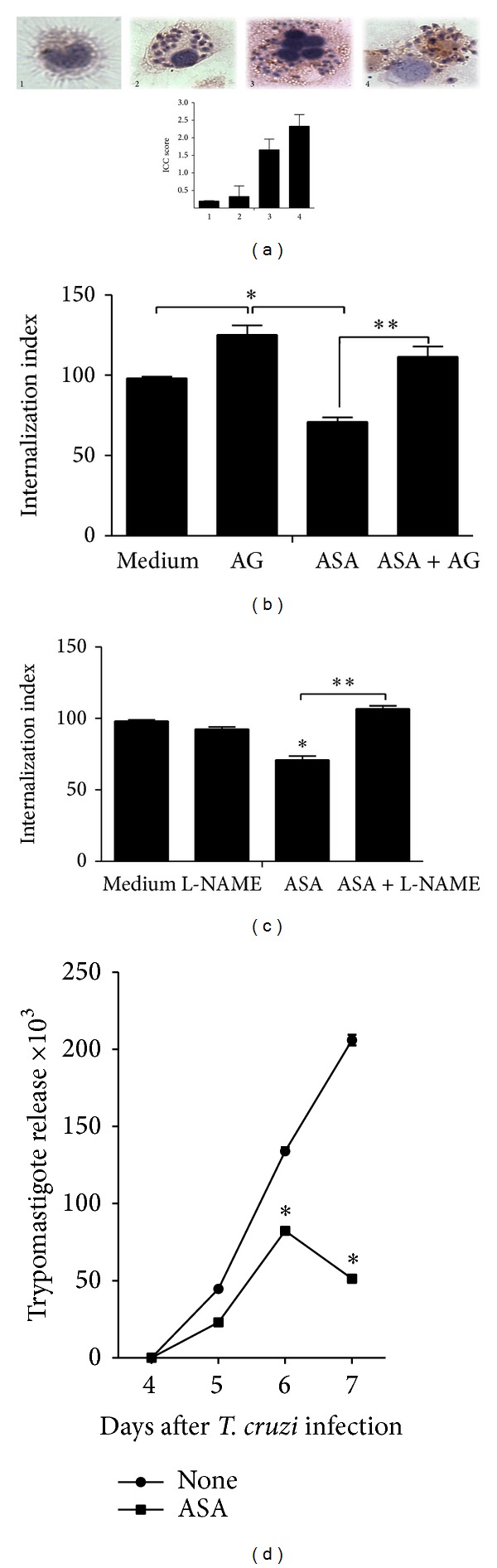
Effect of ASA on macrophage activity depends on NO^∙^ production. (a) Aspirin treatment stimulated iNOs expression in* T. cruzi*-infected macrophages. Immunocytochemistry for iNOS was performed on coverslip-adherent cells using the labeled streptavidin biotin method with a LSAB KIT (DAKO Japan, Kyoto, Japan) without microwave accentuation. (1) Intracellular iNOS protein cannot be detected by immunocytochemistry in uninfected (control) macrophages, (2)* T. cruzi*-infected cell, (3) ASA (0.625 mM) was an effective inducer of iNOS expression in peritoneal macrophages, and (4) the addition of PGE_2_ (3.52 ng mL^−1^) to culture media increased iNOS mRNA expression in macrophages infected. Macrophages were treated for 30 minutes separately with 0.625 *μ*M ASA. After treatment, macrophages were washed and incubated with aminoguanidine (AG, 1.0 mM) (b) or L-NAME (c) at 1.0 mM for 1 h at 37°C. After treatment, macrophages were washed again and interacted with 5 : 1 trypomastigotes for 2 hours at 37°C, after which they are washed, fixed with Bouin's fixative, and stained with Giemsa. Quantification was carried out under a light microscope where the number of intracellular parasites was counted in a total of at least 500 cells. Results are the mean ± standard error for triplicate determinations and are representative of two independent experiments. (d) Effect of aspirin upon trypomastigote release in aspirin-treated* T. cruzi*-infected macrophages. Cells were infected with* T. cruzi* trypomastigotes and treated daily with ASA at 0.625 mM; after 4 days of treatment, trypomastigotes release to supernatants was found and was measured until day 7 after infection. Results are the mean ± standard error for triplicate determinations and are representative of two independent experiments. *P < 0.01 for a comparison with infected cells cultured in medium alone. **P < 0.01 for a comparison with infected cells cultured treated with ASA.

In addition, we observed that ASA treatment stimulated iNOS expression in* T. cruzi*-infected macrophages (Figure 4(a)). To confirm that the effect of ASA on macrophage activity depends on NO^∙^ levels, we assessed the entry of trypomastigotes into macrophages incubated with aminoguanidine (AG, iNOS inhibitor) or with L-NAME (c-NOS inhibitor). We found that both inhibitors reversed the effects of ASA (Figures 4(b) and 4(c)).

## 4. Discussion

Previous studies have shown that the release of eicosanoids during infection with* T. cruzi* regulates host responses and controls disease progression [3, 10, 11, 13, 14, 28–31]. PGs, together with NO^∙^ and TNF-*α*, participate in a complex circuit that controls lymphoproliferative and cytokine responses in* T. cruzi* infection [10]. However, the involvement of COX-mediated PG production in the entry of* T. cruzi* into macrophages is largely unexplored. The data shown herein demonstrates that the treatment of macrophages with ASA significantly inhibits internalization of* T. cruzi* trypomastigotes and strongly supports the idea that the COX pathway plays a fundamental role in the process of parasite invasion. In fact, PGE_2 _production increases significantly in* T. cruzi*-infected macrophages as compared to uninfected macrophages [32] and synergistically enhances the activity of nifurtimox and benznidazole on infected RAW 264.7 cells [23].

Ours results suggest that the actions of ASA depend on COX-2-derived biosynthesis of products. This is demonstrated by the observation that celecoxib reversed the aspirin-induced inhibition of entry of* T. cruzi* into macrophages. The problem of NSAIDs coadministration is actively discussed in literature in the context of uncertainty of the resulting therapeutic and side effects arising from such combinations. The mechanism of such a suppression of aspirin inhibitory effect on COX-1 by other NSAIDs has been difficult to satisfactorily explain. This could be due to celecoxib binding strongly to a monomer of COX-1 without affecting AA oxygenation [7]. In fact, a related study has shown that celecoxib prevented ASA inhibition in a dog model of thrombosis [33]. However, the application of our approach to investigate the combined effect of aspirin and celecoxib on COX-1 during coadministration confirmed the ability of celecoxib to suppress the aspirin-mediated inhibition of COX-1* in vitro* conditions [7].

In addition, the effects of ASA on* T. cruzi* infection have been associated in part, to a switch to the AA pathway linked to the acetylation of the COX-2 isoenzyme [18]. Such modification promotes the synthesis of the 15-R-HETE intermediate, which can be transformed by 5-lipooxygenase to 15-epi-LXA4, a lipid involved in the resolution of inflammation [34]. Accordingly, we assessed the effect of Boc-2 (a specific antagonist of the 15-epi-LXs) on low doses of ASA, indicating that 15-epi-LXA4 is probably involved in the inhibitory effect exerted by ASA on the internalization of* T. cruzi* by macrophages.

Interestingly, when macrophages were treated with PGE_2_ concentration as high as 3.52 ng mL^−1^, we observed a reduction in the entry of parasites, but when we used low concentration of PGE_2_ (0.35 ng mL^−1^), the entry of* T. cruzi* was similar to that observed in untreated macrophages. In addition, we found reversal of ASA effect, even when PGE_2 _concentration as low as 3.52 ng mL^−1^ was used, indicating that PGE_2_ has an important role in ASA effect. Inhibition of COX activity may increase NO^∙^ levels, thus restoring the antiparasitic activity of macrophages [23]. Our results are in agreement with this hypothesis. To confirm that the effect of ASA on macrophage activity depends upon restoring NO^∙^ levels, we assessed the invasive capacity of* T. cruzi* when cells were incubated with aminoguanidine (AG), an iNOS inhibitor. We found that 1.0 mM AG reverses ASA effect totally. In addition, we showed that iNOS expression in macrophages was increased with ASA treatment, suggesting that iNOS-dependent NO^∙^ production is responsible for ASA effects. We did find reversal of ASA effect with L-NAME (1.0 mM), indicating the role of cNOS in ASA activity. This can be explained as high concentrations of L-NAME may interfere in the selectivity for cNOS and can inhibit other isoforms of NOS, such as nNOS and iNOS. So, NO^∙^ deficiency induced by L-NAME could be explaining our results.

Additionally, polyamines seem to be crucial for trypomastigote internalization process in, at least, some cellular types and infection progression [35]. In* T. cruzi*-infected macrophages, COX is related to the increase of ornithine decarboxylase (ODC) activity [15], which might increase the polyamine content in macrophages. Since* T. cruzi* uses these polyamines to synthesize trypanothione (an enzyme that participates in the hydroperoxide detoxification of* T. cruzi*), the inhibition of COX by ASA probably resulted in a reduction in polyamine levels caused by inhibition of ODC, indirectly contributing to decrease trypanothione synthesis in* T. cruzi*, as suggested by López-Muñoz and collaborators [23].

Finally, in* T. cruzi*-infected macrophages, COX inhibition by ASA was related to the increase of IL-1*β*, which also might explain the increase of antiparasitic activity of macrophages treated with ASA. In fact, IL-1*β* is critical for the restriction of* Leishmania amazonensis *infection [36] and it recently was demonstrated that macrophages treated with IL-1*β* released fewer trypomastigotes than untreated macrophages and IL-1*β* triggered NO^∙^ release by* T. cruzi*-infected macrophages in a dose dependent manner [37].

## 5. Conclusion

In conclusion, this is the first report, to our knowledge, showing the* in vitro* effect of aspirin on* T. cruzi *entry into peritoneal macrophages and the influence of COX pathway on innate inflammatory response to* T. cruzi* infection, adding a new perspective to immune interventions against Chagas disease.
